# Modelling Pathways to Rubisco Degradation: A Structural Equation Network Modelling Approach

**DOI:** 10.1371/journal.pone.0087597

**Published:** 2014-02-03

**Authors:** Catherine Tétard-Jones, Angharad M. R. Gatehouse, Julia Cooper, Carlo Leifert, Steven Rushton

**Affiliations:** 1 Molecular Agriculture Group, Nafferton Ecological Farming Group, School of Agriculture, Food and Rural Development, Newcastle University, Newcastle-Upon-Tyne, United Kingdom; 2 Molecular Agriculture Group, School of Biology, Newcastle University, Newcastle-Upon-Tyne, United Kingdom; 3 School of Biology, Newcastle University, Newcastle-Upon-Tyne, United Kingdom; Memorial Sloan Kettering Cancer Center, United States of America

## Abstract

‘Omics analysis (transcriptomics, proteomics) quantifies changes in gene/protein expression, providing a snapshot of changes in biochemical pathways over time. Although tools such as modelling that are needed to investigate the relationships between genes/proteins already exist, they are rarely utilised. We consider the potential for using Structural Equation Modelling to investigate protein-protein interactions in a proposed Rubisco protein degradation pathway using previously published data from 2D electrophoresis and mass spectrometry proteome analysis. These informed the development of a prior model that hypothesised a pathway of Rubisco Large Subunit and Small Subunit degradation, producing both primary and secondary degradation products. While some of the putative pathways were confirmed by the modelling approach, the model also demonstrated features that had not been originally hypothesised. We used Bayesian analysis based on Markov Chain Monte Carlo simulation to generate output statistics suggesting that the model had replicated the variation in the observed data due to protein-protein interactions. This study represents an early step in the development of approaches that seek to enable the full utilisation of information regarding the dynamics of biochemical pathways contained within proteomics data. As these approaches gain attention, they will guide the design and conduct of experiments that enable ‘Omics modelling to become a common place practice within molecular biology.

## Introduction

Transcriptome and more recently proteome analysis are capable of producing vast amounts of data that provide snapshots of changes in gene expression through time in relation to developmental or environmental variation [1]–[10]. This has been fuelled by technological developments in the last decade that enable large sample throughput and expression analysis of a growing number of species. Whilst much of this work has been developed in model species such as Arabidopsis [3], [10], [11], there is increased interest in species of applied significance such as crops [4], [6], [12], [13]. However, extracting biological meaning from the abundance of ‘Omics data can be limited by a shortfall in the uptake of tools such as modelling [14], [15]. One of the central goals of ‘Omics is to understand the biochemical pathways and networks that link environmental changes to phenotypic effects. The standard presentation of an ‘Omics study is to compare differential gene expression under contrasting environmental conditions, with the assumption that the two are causally linked. Similarities in the physiological function of differentially expressed genes/proteins may then indicate the biochemical pathways involved. Progress towards describing the biological process requires inference and modelling to assess the significance of likely relationships. Several studies that have initiated a movement towards integrating modelling tools with ‘Omics data have tended to use single celled organisms and well characterised systems as an exploration of modelling capabilities [14], [16]–[18]. An important challenge is to test these modelling tools with ‘Omics datasets for previously uncharacterised or descriptive whole organism systems, to enable modelling to become standard practice of ‘Omics data analysis.

Proteomics methods (including 2-D electrophoresis (2DE) and mass spectrometry (MS)) are particularly important for detecting and identifying post-translational modifications (PTMs). PTMs can have an important role in plant physiology, for example the degradation of the multi-subunit protein Rubisco. Rubisco has a crucial role in carbon fixation, but is relatively inefficient and limits the rate of photosynthesis. Therefore, the structure and function of Rubisco and its genes receives much attention to understand how its efficiency in carbon fixation could be improved, which would have a dramatic impact on crop production [19], [20]. Rubisco is also involved in a second important plant process – nitrogen remobilisation [20], [21]. Due to the inefficiency of Rubisco, plants need to produce high levels of it in order to photosynthesise. Consequently, a high level of plant nitrogen (up to 30%) is contained within total plant Rubisco and is therefore an important component of a plants’ N budget for growth and seed production [22]. In cereal crops such as wheat, nitrogen does not permanently remain in leaves. Nitrogen is used for the synthesis of proteins such as Rubisco in young growing leaves. Wheat leaves start to senescence shortly after leaf elongation, synchronised with the growth of new leaves higher up the plant. Proteins in the senescing leaf are degraded, to enable the remobilisation and transportation of nitrogen to the young leaf for the synthesis of new Rubisco. Rubisco is thought to be degraded in two cellular compartments; degradation can initially occur within the chloroplast by reactive oxygen species (ROS) that make it more susceptible to peptide hydrolases (e.g. metaloendopeptidases), which produces a Rubisco fragment that has increased infinity to bind to the chloroplast envelope to be transported out of the chloroplast [23]. Outside the chloroplast, Rubisco fragments can be contained within specific bodies (Rubisco containing bodies) within vacuoles or in the cytoplasm [24] where the Rubisco fragments can be further degraded by vacuolar cysteine endopeptidases [25]. Rubisco nitrogen is eventually transported from the senescing tissue in the form of peptides or amino acids via the phloem to young growing leaves or seeds. Despite the appearance of well-regulated pathways of protein degradation for N remobilisation, it is not 100% efficient. In wheat, approximately 80–90% of plant N uptake is eventually remobilised into the grain, depending on wheat variety and crop management factors [26], [27]. Nitrogen that has not been transported out of the leaf prior to death remains in the tissue and therefore cannot be used in any further growth or grain production. Understanding the factors that affect the efficiency of nitrogen remobilisation, particularly the dynamics of the degradation process of nitrogen rich Rubisco, may aid attempts to increase the efficiency of nitrogen remobilisation via crop breeding and optimisation of production regime [23]. For example, environmental factors such as N deficiency can increase the efficiency of nitrogen remobilisation, however it is not clear how plants regulate the efficiency of this process [27], [28]. To understand the dynamics of the protein degradation process there is need for a statistical modelling approach that makes testable predictions and tests hypotheses regarding the relationships between the protein and its degradation products. This would address important questions such as how many stages of degradation does Rubisco go through and how the degradation process is affected by the proteins’ environment (e.g. plant nutritional status).

In this study we consider the potential for using Structural Equation Modelling (SEM) to investigate protein-protein interactions in a proposed Rubisco protein degradation pathway using data from Tétard-Jones et al [27]. SEM investigates relationships between different processes by partitioning them among variables on the basis of a hypothetical pathway of interaction that are identified *a priori*. Paths between variables are defined in equation form, where response variables can be related to multiple predictor variables and response variables in one prediction can form the predictor in others. SEM uses the variance and covariance matrix to test whether the variables in the path are interrelated. The simplest and best explanation model for the available data is then identified from goodness-of-fit criteria. Whilst SEM has been used in the analysis of a wide range of areas from ecological through to medical contexts [29]–[32], its use in ‘Omics analysis has been less frequent [33] particularly in proteomics. A more frequently used approach in ‘Omics use Bayesian methods [17], [34]. Combining Bayesian methods with SEM (Bayesian estimation of SEM) enables the use of prior information, improving parameter and latent variable estimates, statistics for model comparison and provides more reliable results for datasets that are based on ‘small’ sample sizes as is typical of ‘Omics experiments [18], [35].

We used the Rubisco degradation process as a test system to trial a novel framework for modelling protein degradation processes based on the available data from 2DE and MS proteome analysis [36]. The combination of 2DE and MS produces data for both protein expression and protein sequence, which adds a physical reality to the statistical model output connecting degradation products to the native/intact protein. This study represents an early step in the development of approaches that seek to enable the full utilisation of information regarding the dynamics of biochemical pathways contained within proteomics data.

## Methods

### Development of a Prior Model

**Figure 1:**
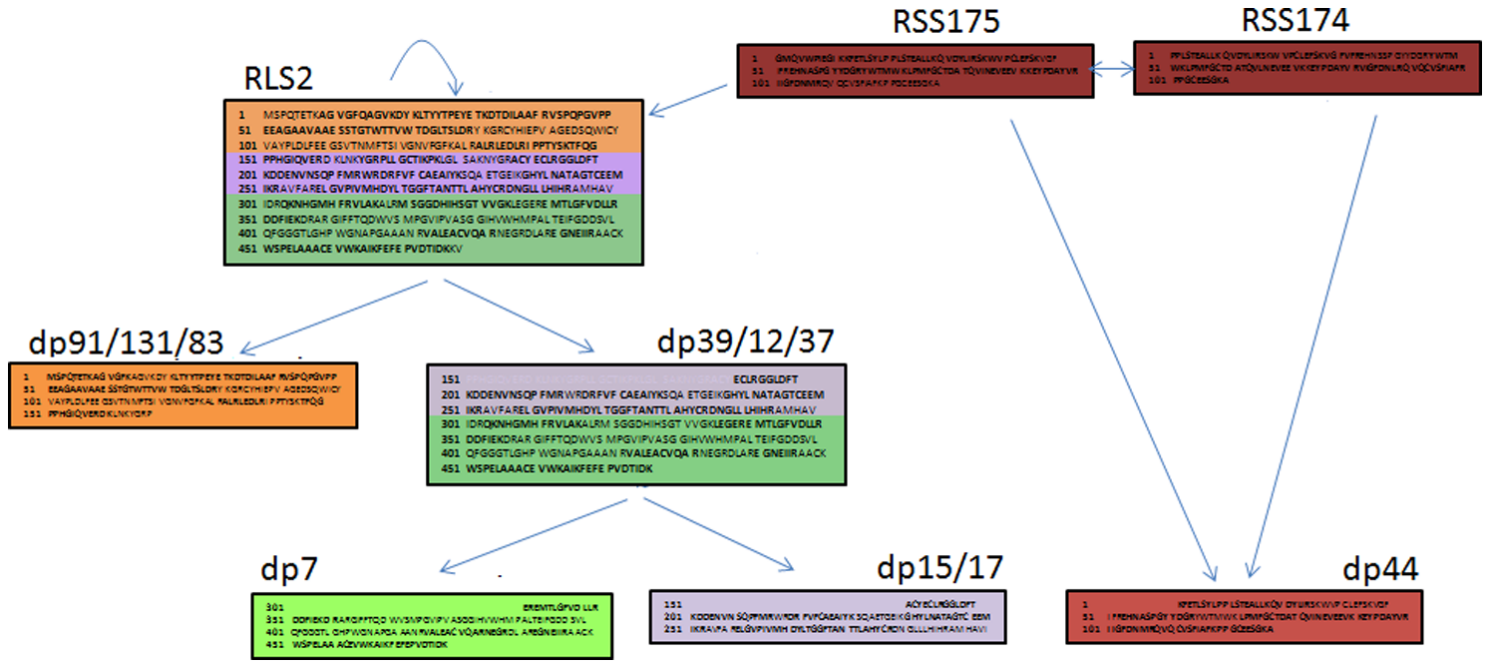
Hypothetical model of Rubisco Large Subunit (RLS2) and Small Subunit (RSS#) degradation generated by using prior information. The coloured text boxes indicate identical protein sequences in the intact proteins and the degradation products (dp#). Please see Tétard-Jones et al. 2013 [27] for detailed information of the protein sequences. Arrows connect proteins together that are assumed to have a direct relationship (e.g. dp44 is a degradation product of RSS174 and/or RSS175).

We developed a prior model of the Rubisco degradation process involving all intact Rubisco subunits and their degradation products (Fig. 1). This model was based on several pieces of information from a published study that had used 2D electrophoresis (2DE) and mass spectrometry (MS) to detect changes in the wheat leaf proteome at three time points [36]. Protein identification and mass allowed us to separate intact Rubisco subunits from its degradation products. Protein sequence information (from MS) enabled the placement of degradation products in a conceptual stepwise pathway. We assumed that if a degradation product contained a portion of the whole protein sequence of a particular Rubisco subunit (either Rubisco Large Subunit (RLS) or Small Subunit (RSS)), this indicated a link between that intact subunit and the primary degradation product. Similarly, if a degradation product contained a smaller portion of the same protein sequence, then this indicated a link between the primary and secondary degradation product (i.e. a further degradation product of a degradation product). In addition, we included links based on information from another published study showing that production of Rubisco Large Subunit (RLS) can be regulated by feedback (self-regulation when in excess) and up-regulated by the presence of Rubisco Small Subunit (RSS) [37].

### Testing the Validity of the Prior Model

Structural Equation Modelling (SEM) was used to investigate the relationships among the intact Rubisco subunits and their degradation products in the prior model. The model was tested for each degradation product and possible relationship with Rubisco subunits, whilst non-significant pathways were removed. The presence of latent variables, composed of proteins that had near identical expression (multi-collinearity) was also tested. The level of accuracy for the model to explain variation in the data due to relationships between variables (correlations or covariances) is termed “goodness of fit”, for which several indexes are available. In our analysis, Goodness of fit was assessed using i) the root mean square error of approximation (RMSEA), which should be <0.1 with a p-value >0.05 and a 90% confidence interval 0.05–0.10, ii) Standardised Root Mean Square Residual, and iii) consideration of the significance of the individual path coefficients. Whilst the fit indexes were used to evaluate the accuracy of the model-data fit, the physical reality of the protein mass and sequence data were used to guide alterations to the model. The SEM models were fitted using maximum likelihood (ML) estimation in R lavaan package. Small sample size (<200, or <10x the number of connections between variables in the model) is a problem for acquiring valid statistical inference from SEM. Therefore we were only able to use SEM to indicate model structure. This is a useful first step in modelling before adopting a Bayesian estimation of SEM [18], [35], [38].

The best fit model from SEM was translated into a Bayesian model in Jags in R (rjags package). Jags uses Markov Chain Monte Carlo (MCMC) simulation to generate a posterior distribution of the model parameters (variables) that is tested for convergence, i.e. whether the model explains the distribution of the observed data [39]. We used the Gelman-Rubin convergence diagnostic, which measures the difference within several chains and the variance between several chains of the MCMC simulation by the potential scale reduction factors (psrf). Convergence is met when the output from all chains is indistinguishable. The R code for both the SEM and Jags models are provided in Supporting Information Text S1 and Text S2.

## Results

### Rubisco Degradation Model Validation using Structural Equation Modelling (SEM) and Bayesian Estimation

**Figure 2:**
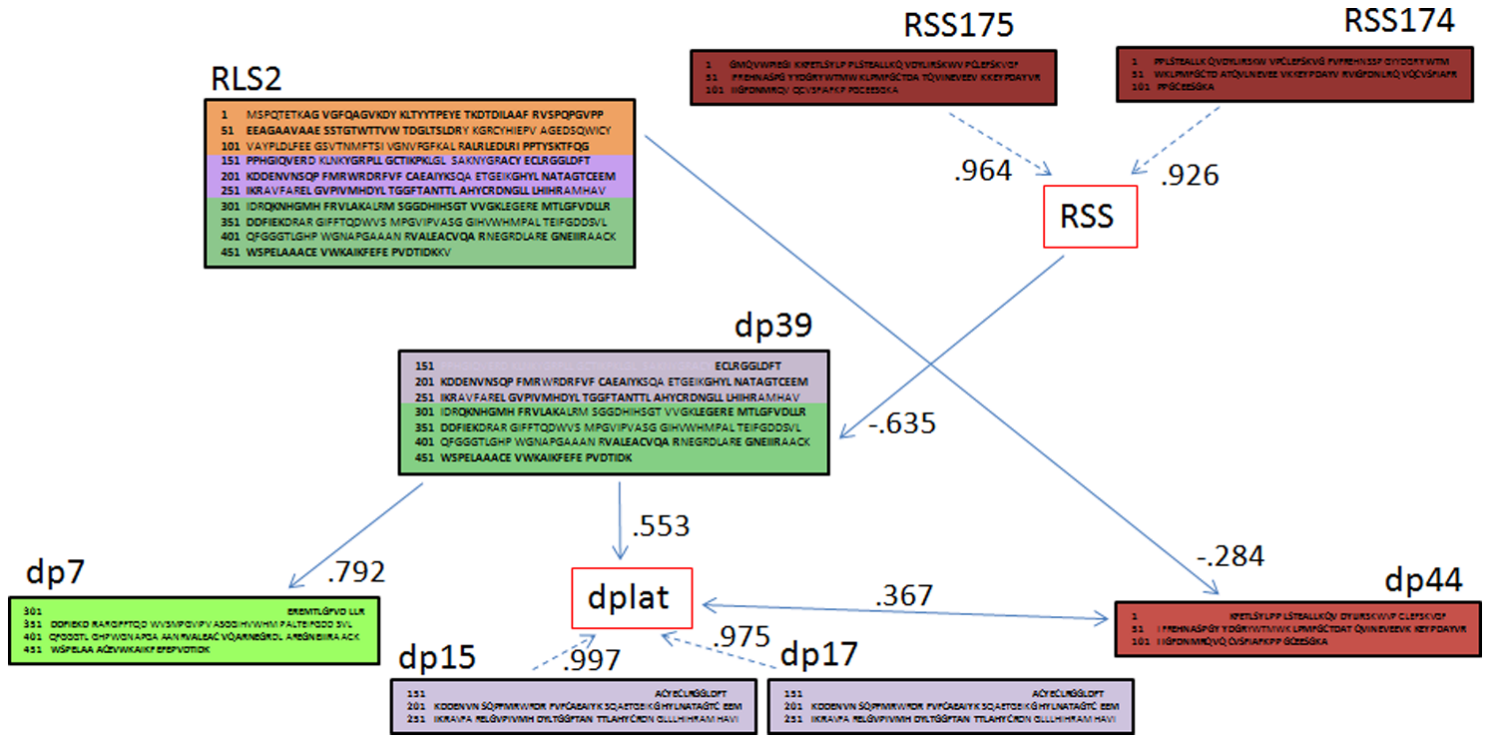
Model of Rubisco degradation following Bayesian estimation of SEM to test the hypothetical model. The coloured text boxes indicate identical protein sequences in the intact proteins and the degradation products (dp#). Please see Tétard-Jones et al. 2013 [27] for detailed information of the protein sequences. Decimal figures indicate the standardised coefficients for the relationship indicated by the arrows.

**Figure 3:**
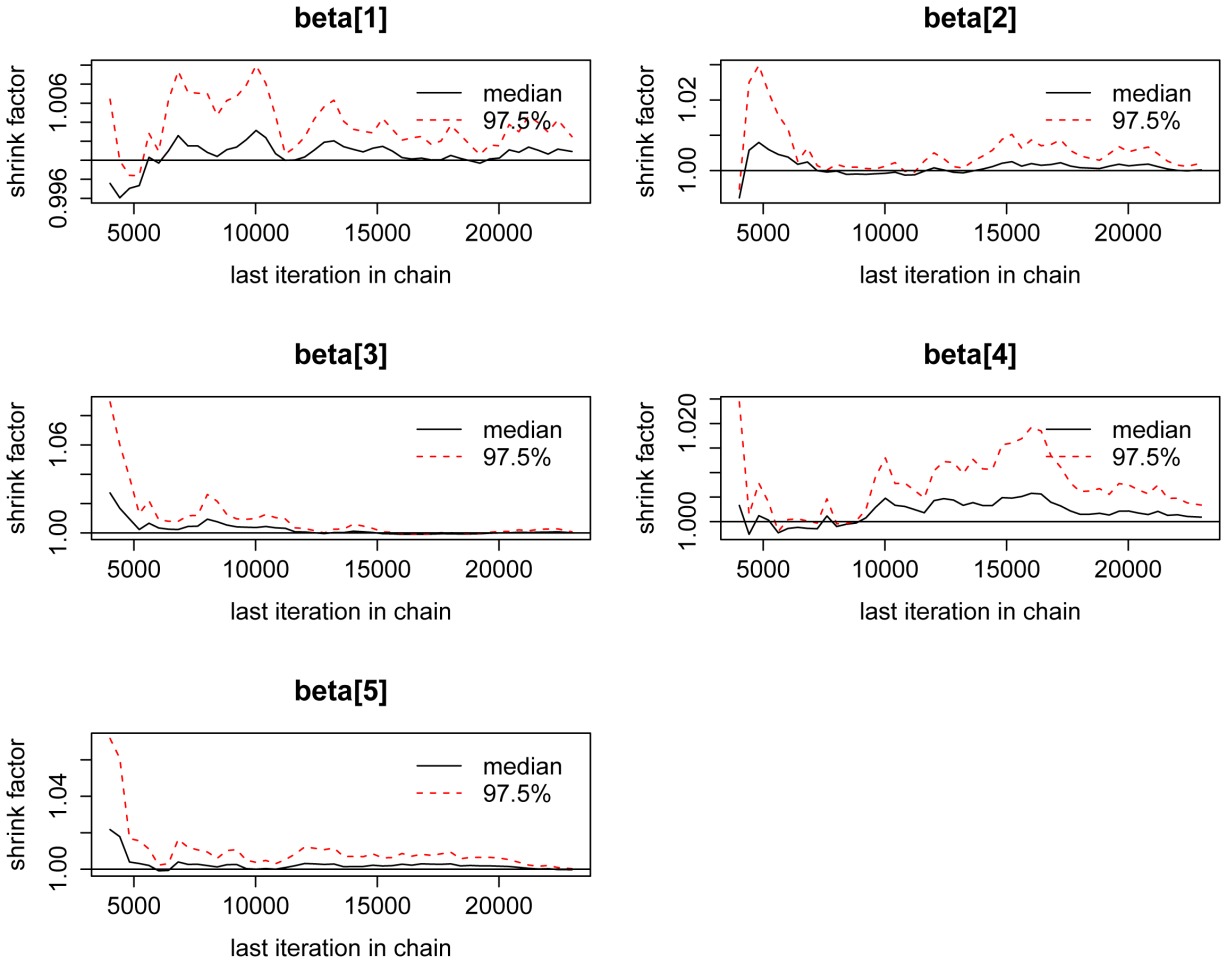
Gelman-Rubin plots. These plots show the development of the potential scale reduction factors (psrf) for modelled parameters over 20,000 iterations of 4 chains. Multivariate psrf = 1.

We initiated model validation with the observed data using SEM to investigate relationships between intact Rubisco subunits and their degradation products. After several rounds of model adjustment and model testing, we obtained a ‘good fit’ that converged normally after 109 iterations; RMSEA (Root Mean Square Error of Approximation) 0.082, 90% confidence interval: 0.000 0.164, p-value RMSEA 0.269, SRMR (Standardised Root Mean Square Residual) 0.100. The removal of non-significant pathways greatly improved the model fit, and the final model with all of the non-significant variables removed is shown in figure 2, with standardised coefficients for each of the modelled relationships. Testing for multi-collinearity (correlation <0.9) identified two latent variables, which represented the two Rubisco Small Subunits (RSS) and two of the secondary degradation products (dplat). This SEM model was then translated into Jags (full R code with parameters are provided in Supporting Information Text S1 and Text S2) following Song and Lee [18], [35]. To test whether the model explained the variance observed in the data we used the Gelman-Rubin convergence diagnostic. If convergence is achieved, then all chains in the Markov Chain Monte Carlo (MCMC) simulation should be indistinguishable, i.e. converge. This can be visualised with the development of the potential scale reduction factors (psrf) over the chain iterations (figure 3). We obtained a multivariate psrf of 1 indicating that complete convergence was achieved for all of the model parameters and that the model had explained the variance observed in the data.

### Bayesian Estimation of a SEM Model for Rubisco Degradation

The hypothesised relationship between Rubisco Large Subunit (RLS) degradation products dp39 to dp7 and dp15 and dp17 was confirmed, indicating that Rubisco is degraded in a stepwise process (rather than all degradation products produced from the intact RLS at the same time). By testing for multi-collinearity (correlation >0.9) we identified a latent variable composed of dp15 and dp17, as well as a second latent variable for the Rubisco Small Subunits (RSS174 and RSS175). The main difference between the prior and actual models was the relationship between the intact Rubisco subunits (RLS2 and RSS) and the degradation products. We had assumed (based on protein sequence data) that degradation products would be most strongly related to proteins that they were products of, i.e. RLS2– dp39/12/37 and RSS – dp44. However, these pathways were not significant, and the observed data indicated significant pathways between RLS2 and dp44 (RSS degradation product) and RSS with RLS2 degradation products. Although counter intuitive, this may be due to the time points that these proteins were measured. If they were too far apart in time, the data may have captured indirect protein – degradation product relationships. For example in the original proposed pathway of RSS-RLS2-dp39 we may have missed the direct links with RLS2 leaving RSS-dp39 as a remnant of this pathway.

## Discussion

‘Omics technology is capable of producing vast amounts of data that provide snapshots of gene expression during a time series that tracks changes in an organism’s phenotype associated with variation in the environment [1]–[10]. ‘Omics aims to understand the molecular pathways that form such connections; however this has tended to be limited by the lack of uptake of tools such as modelling [14], [15]. Modelling approaches allow the investigation of relationships between expressed genes, building pathways or networks of biological importance. The degradation of Rubisco is a process that is important in plants for the remobilisation of the proteins high nitrogen content [20]–[23]. Despite this importance, little is understood regarding its degradation pathway – for example how many degradation products are produced, and over how many levels (e.g. primary, secondary). To understand the dynamics of protein degradation processes there is need for a statistical modelling approach that makes testable predictions and challenges hypotheses regarding the relationships between the protein and its degradation products. In this study, we applied a combined SEM and Bayesian modelling approach to a previously published dataset for Rubisco and some its degradation products to investigate whether such approaches provided greater insight into the dynamics of protein degradation. As well as providing biologically interesting findings, this was also an opportunity to consider the potential for using SEM to investigate protein-protein interactions in a dataset generated by 2D electrophoresis (2DE) and mass spectrometry (MS) proteomics technology.

This study highlighted one of the benefits of using 2DE and MS generated data – that it provides protein mass and sequences, which can add a physical reality to the statistical modelling output for a protein degradation pathway. This prior information provided the foundation for the hypothetical model, which identified a pathway model constructed of intact protein subunits and both primary and secondary degradation products. Our modelling approach initially used SEM to test relationships between proteins as proposed in the prior model. SEM operates by testing whether the variables in a path are interrelated by analysing their variances and covariances. This makes SEM an ideal tool to test for variables that are latent due to multi-collinearity (i.e. their expression is near identical across time or environmental variation). We identified two latent variables due to multi-collinearity, which was also physically confirmed by the identical protein mass and near identical protein sequences for the variables within each latent. The RSS latent variable indicates that the expression of the two Rubisco Small Subunits is regulated by the same mechanism. Similarly the two distinct secondary degradation products of Rubisco Large Subunit (RLS) that contributed to dplat appear to be co-expressed (in this case co-produced by the degradation of dp39).

SEM can also have limitations for modelling ‘Omics data, due to its reliance on datasets with a large number of samples (100–200 samples). Sample size is a problematic issue in SEM based studies. The number of samples required to support the credibility of research conclusions depends on model complexity. Model complexity refers to how many links there are between parameters compared to the number of parameters, and may include multiple feedback links and also the number of latent variables. Generally the greater the model complexity, the more observations are needed. Therefore sample size requirement in SEM depends on the dynamics of the model, which may be unknown until the data has been collected and statistically analysed. Existing guidance for setting sample size in SEM based studies recommends a minimum of 100–200 samples [40], and several algorithms are available online and in R to calculate the precise sample size required depending on model complexity. In our study that consisted of 48 samples, we observed the problem of small sample size in the RMSEA goodness of fit index. Our 90% confidence interval (0.00–0.162) indicated that the model had close approximate fit to the data, shown by the lower bound of the interval (<0.05). However, the upper bound was above the 0.100 cut-off value, which indicates poor fit. Thus the overall RMSEA was 0.082, which indicates a mediocre fit (0.01 = excellent fit, 0.1 = poor fit). These RMSEA values show that the model contains sampling error. This outcome is typical of datasets with small samples, and displays one of the major limitations of SEM when used as the sole modelling tool [40]. In summary, SEM indicated that we had a model with good fit to the data, but our sample size was too small for SEM to provide any valid statistical inference. In comparison, Bayesian analysis incorporating MCMC simulation provides accurate parameter estimates regarding how well the model replicates the variation in data consisting of small sample sizes [38]. This approach has been used in several studies using single celled organisms and well characterised systems [14], [16]–[18]. MCMC simulation avoids the problem of small sample size by running the model several times, known as ‘chains’. The model receives prior information (i.e. information from our prior model) and then fits this model to the data, in our case for 4 chains. Goodness-of-fit is diagnosed using convergence diagnostics (for example the Gelman-Rubin used here), which indicate when the output of all the chains has converged – the output of each chain is indistinguishable from the others.

Our approach to modelling a Rubisco degradation pathways using data from 2DE and MS proteomics analysis [36] identified features not previously reported. This included the RSS degradation product (dp44), and secondary RLS degradation products (dplat and dp7) whose link to the primary RLS degradation product (dp39) was confirmed by the model. However, our modelling approach could be criticised on the basis that the data had not originally been intended for a modelling analysis and therefore lacked two ideal attributes. The first attribute is the inclusion of an appropriate time series. To make the model dynamic (i.e. accurately model protein-protein interactions over time) the number of steps in the time series and the interval between steps needs to capture changes in the expression of each protein in fine detail. In this study, we attempted to make the model dynamic (i.e. by creating a different node for each protein at each of our 3 time steps); however we were unable to obtain a good fit. It is likely that this failed because the intervals between time-steps were too far apart, since they had been chosen based on agronomic rationale rather than a proteomic modelling rationale. Recent studies modelling gene expression data using Dynamic Bayesian Networks (DBN) demonstrate models based on well characterised pathways using a time series consisting of around 5–10 time steps separated at intervals spanning minutes, hours or up to days rather than weeks apart [16], [21], [34]. The second attribute that we considered may have limited the model in this study is the possibility that there are proteins involved in the Rubisco degradation pathway that we did not have the data to include in the model. This is likely to include other Rubisco subunits; Rubisco is known to be composed of 8 large subunits and 8 small subunits, whereas we had data for 1 large and 2 small Rubisco subunits. In addition, there may be other degradation products, and also the proteins that degrade Rubisco, which were not available in our data. The lack of data for these proteins is due to the nature of the proteomics analysis that this data originated from. In 2DE proteomics analysis, all soluble proteins are purified, separated and the expression of each protein spot on the 2DE gel is quantified. Only the proteins that are of interest (based on fold changes in expression through time or other experimental parameters) are extracted from the gel and identified by MS (mass spectrometry). Therefore, the proteins included in this study are only those whose expression did significantly alter over the parameters of the original experiment. Studies that design experiments to capture changes in all variables of a pathway or network require prior identification of those important variables, which was not available to inform the study that supplied our data [36]. For this reason, studies that have initiated a movement towards integrating modelling tools with ‘Omics data have tended to use less complex single celled organisms and well characterised systems to explore ‘Omics modelling capabilities [14], [16]–[18]. However, progress towards systems of an applied interest, for example that has agronomic importance, such as wheat is necessary. In conclusion, the model investigated in this study could be described as a simplified model that has captured a Rubisco degradation pathway that may be part of a larger Rubisco degradation network in an agronomically important crop species, wheat. Fully expanded, this could be developed to model a whole plant Rubisco production and degradation network, incorporating remobilisation of nitrogen degraded from senescing leaves, transported to developing leaves (and finally grain) to be re-used in the production of new Rubisco. In addition, the model could be developed to incorporate additional factors involved in degradation-remobilisation at the protein or transcript level (e.g. Rubisco transcripts, metalloendopeptidases, vacuolar cysteine endopeptidases) to capture the dynamics of Rubisco synthesis as well as synthesis of the enzymes that degrade Rubisco. A further application of our proposed ‘Omics modelling approach could be to clarify the extent that Rubisco is degraded in each cellular compartment (chloroplasts and cytoplasm or vacuolar Rubisco containing bodies) and identify the enzymes involved, for example by combining 2D electrophoresis of cellular compartments with a series of appropriate time points tracing the progress of leaf senescence.

In conclusion, this study highlights the potential to integrate ‘Omics analysis with a modelling approach to investigate the relationships between genes or protein within the study system. Without this integration of techniques ‘Omics can make hypotheses concerning molecular pathways supported by snapshots of gene/protein expression, but it is not able to validate whether expression of one gene/protein does in fact affect the expression of other genes/proteins in the same or other pathways. As this area gains momentum the need for such an approach will guide the design and conduct of experiments that enable ‘Omics modelling to become a common place practice within molecular biology to better understand the dynamics of biochemical pathways.

## Supporting Information

Text S1 **SEM model for the lavaan package in R.**
(DOCX)

Text S2 **Jags model for the rjags package in R.**
(DOCX)
