# Supplementary material for: Modelling Pathways to Rubisco Degradation: A Structural Equation Network Modelling Approach
Source: PLoS One. 2014 Feb 3;9(2):e87597. doi: 10.1371/journal.pone.0087597 (PMC3911993; doi:10.1371/journal.pone.0087597)
Supplement: Text S1 — SEM model for the lavaan package in R. (DOCX) [file pone.0087597.s001.docx]

Text S1. SEM model for the lavaan package in R.

#load data

Setwd(“*location of data file*”)

Rawdprot<-read.table(“rawdprot.csv”, header=TRUE, sep=”,”)

library(lavaan)

#load model

sem5.model<-‘

RSS=~RSS175+RSS174

dplat~dp39

dp39~RSS

dp7~dp39

dp44~RLS2

dp44~~dplat

’

#run model

Sem5.model<-sem(sem5.model,data=rawdprot)

Summary(sem5.model, fit.measures=TRUE)
