# Supplementary material for: Modelling Pathways to Rubisco Degradation: A Structural Equation Network Modelling Approach
Source: PLoS One. 2014 Feb 3;9(2):e87597. doi: 10.1371/journal.pone.0087597 (PMC3911993; doi:10.1371/journal.pone.0087597)
Supplement: Text S2 — Jags model for the rjags package in R. (DOCX) [file pone.0087597.s002.docx]

Text S2. Jags model for the rjags package in R.

#load data

Jags6.data<-list(RLS2=rawdprot$RLS2,

RSS175=rawdprot$RSS175,

RSS174=rawdprot$RSS174,

dp39=rawdprot$dp39,

dp7=rawdprot$dp7,

dp17=rawdprot$dp17,

dp15=rawdprot$dp15,

dp44=rawdprot$dp44,

X=nrow(rawdprot))

#load model

cat("model

{

for(i in 1:X)

{

RSS[i] <- RSS175[i] + RSS174[i]

dp39.hat[i]<-beta[1]*(RSS[i] - mean(RSS[]))

dp39[i] ~ dnorm(dp39.hat[i], tau.u[1])

dp17.hat[i]<-beta[2]*(dp39[i] - mean(dp39[]))

dp17[i] ~ dnorm(dp17.hat[i], tau.u[2])

dp15.hat[i]<-beta[3] *(dp39[i] - mean(dp39[]))

dp15[i] ~ dnorm(dp15.hat[i], tau.u[3])

dp7.hat[i]<-beta[4] *(dp39[i] - mean(dp39[]))

dp7[i] ~ dnorm(dp7.hat[i], tau.u[4])

dp44.hat[i]<-beta[5] *(RLS2[i] - mean(RLS2[]))

dp44[i] ~ dnorm(dp44.hat[i], tau.u[5])

}

#priors

#fixed intercept and slope

beta[1]~dnorm(0,0.0001)

beta[2]~dnorm(0,0.0001)

beta[3]~dnorm(0,0.0001)

beta[4]~dnorm(0,0.0001)

beta[5]~dnorm(0,0.0001)

#residual error

tau.u[1]<-pow(sigma[1], -2)

sigma[1] ~ dunif(0,100)

tau.u[2]<-pow(sigma[2], -2)

sigma[2] ~ dunif(0,100)

tau.u[3]<-pow(sigma[3], -2)

sigma[3] ~ dunif(0,100)

tau.u[4]<-pow(sigma[4], -2)

sigma[4] ~ dunif(0,100)

tau.u[5]<-pow(sigma[4], -2)

sigma[5] ~ dunif(0,100)

}",

file="Jag6.jag")

#run jags

library(rjags)

jags6.out <- jags.model('Jag6.jag',

data=Jags6.data,

n.chains=4,

n.adapt=1000)

update(jags6.out, 1000)

#generate posterior samples

jags.samples(jags6.out,

c('beta'),

1000)

# getting results

Jags6.res<-coda.samples(jags6.out,

var=c("beta"), "sigma"

n.iter=20000,

thin=20)
